# Antioxidant potential and genomic adaptation of *Cetobacterium ceti* MaLMAid0298 from the gills of *Sebastiscus marmoratus*

**DOI:** 10.3389/fmicb.2026.1815687

**Published:** 2026-06-25

**Authors:** Jun Won Lee, Jihyun Yu, Yeon-Ju Lee, Hyein Jin, Jung Eun Yang, Kae Kyoung Kwon, Yun Jae Kim

**Affiliations:** 1 Marine Biotechnology Research Center, Korea Institute of Ocean Science and Technology, Busan, Republic of Korea; 2 KIOST School, University of Science and Technology, Daejeon, Republic of Korea; 3 Marine Natural Products Chemistry Laboratory, Korea Institute of Ocean Science and Technology, Busan, Republic of Korea; 4 Department of Biological Application and Technology, National Marine Biodiversity Institute of Korea, Seocheon, Republic of Korea; 5 Gyeonggido Business and Science Accelerator, Suwon, Republic of Korea

**Keywords:** antioxidant activity, *Cetobacterium ceti*, deep learning, fish microbiome, genome mining

## Abstract

*Cetobacterium* species are common members of fish-associated microbiomes and have been reported from diverse hosts, ranging from freshwater fishes such as carp and tilapia to marine sciaenids. However, their genomic characteristics and roles in oxidative stress defense remain poorly understood. In this study, we present a comprehensive characterization of *Cetobacterium ceti* strain MaLMAid0298, isolated from gill of the false kelpfish *Sebastiscus marmoratus*. A high-quality complete genome was assembled using a hybrid approach combining Illumina and Nanopore sequencing, and phylogenomic analysis confirmed its placement within the *C*. *ceti* clade. Genomic annotation revealed a thioredoxin-centered redox system adapted to anaerobic environments and complete pathways for cobalamin (vitamin B12) biosynthesis. To further explore its antioxidant potential, we established a genome-wide screening pipeline. Candidate peptides were prioritized using deep learning–based activity prediction models. Although the crude extract exhibited limited direct radical scavenging activity in cell-free assays (DPPH and ABTS), it demonstrated significant intracellular reactive oxygen species (ROS) inhibition (52.43%). These findings provide an integrated genomic and functional view of the antioxidant capacity of *C*. *ceti* MaLMAid0298.

## 1 Introduction

Marine fish harbor complex microbial communities within their gastrointestinal tracts, gills, and mucosal surfaces (Merrifield and Rodiles, 2015; Tarnecki et al., 2017). These microbiomes have symbiotic relationships that are essential for host physiology, habitat, and ecosystem stability (Sylvain et al., 2020; Sadeghi et al., 2023). Similar to the well-characterized microbiomes of mammals, fish-associated microbiota contribute to nutrient metabolism, vitamin biosynthesis, and immune modulation, thereby influencing host growth, stress tolerance, and overall health (Butt and Volkoff, 2019; Ou et al., 2021). Many fish microbiomes remain poorly understood, and numerous host-associated bacterial taxa and their functional roles are yet to be characterized (Talwar et al., 2018). Consequently, these microbial resources are increasingly recognized as reservoirs of bioactive strains with potential applications in diverse biotechnological fields (Sanchez et al., 2012). For example, dietary delivery of the fish-associated probiotic strain *Shewanella putrefaciens* Pdp11 was reported to improve immune responses, stress tolerance, and disease resistance in *Senegalese sole* (Tapia-Paniagua et al., 2014). Environmental stressors such as temperature fluctuation, hypoxia, salinity change, pathogens, and pollutants can induce oxidative stress in fish (Birnie-Gauvin et al., 2017), highlighting the potential role of antioxidant-producing bacteria in host–microbe symbiosis.

Among the bacterial genera inhabiting fish intestines, *Cetobacterium* species were discovered as dominant members of freshwater fish microbiomes (Tsuchiya et al., 2008; van Kessel et al., 2011; Larsen et al., 2014) and detected in several marine fish species (Li et al., 2019). Recent studies showed that the *Cetobacterium* is correlated with antioxidant biomarkers (Yang et al., 2023; Zhao et al., 2023) and vitamin B_12_ production (Tsuchiya et al., 2008; Suzuki et al., 2022; Qi et al., 2023). Several studies suggested that *Cetobacterium* plays a protective role within the intestinal environment, enhancing host immunity and epithelial stability (Xie et al., 2022; Qi et al., 2023). However, whether these functions differ among *Cetobacterium* species remains insufficiently resolved. To date, the genus *Cetobacterium* includes *C. ceti, C. somerae*, and the recently proposed fish-derived taxon *Cetobacterium sp*. nov. C33 (Foster et al., 1995; Finegold et al., 2003; Colorado Gómez et al., 2023). Although these taxa have been reported from different host-associated environments, species-level functional differences remain insufficiently resolved, and the physiological and functional characteristics of *C. ceti* in fish-associated habitats remain poorly studied (Suzuki et al., 2022).

Previous studies have associated *Cetobacterium* with antioxidant-related responses in fish, including changes in superoxide dismutase and glutathione peroxidase activity, and radical-scavenging activity has also been reported for *C. somerae* culture supernatants (Yang et al., 2023; Zhao et al., 2023). However, the genomic basis underlying these antioxidant-related traits remains poorly characterized. Recent advances in bacterial genomics and peptide informatics enabled the identification of bioactive sequences directly from genome data (Sekurova et al., 2019; Scherlach and Hertweck, 2021). Such genome-to-peptide approaches allowed for the prediction of short functional fragments with antioxidant, antihypertensive, and antimicrobial activities based on physicochemical properties and deep-learning models (Olsen et al., 2020; Rivero-Pino et al., 2023). These *in silico* analyses may help identify candidate molecular features associated with bacterial adaptation to host-associated oxidative environments. Combining genomic and computational analyses enables efficient identification of functional peptides before experimental validation (Sekurova et al., 2019).

In this study, we present a comprehensive genome-based functional characterization of *Cetobacterium ceti* strain MaLMAid0298, which was isolated from the gill tissue of a marine fish. Whole-genome sequencing and comparative phylogenomic analyses were conducted to determine its taxonomic position and to identify genes associated with antioxidant functions. Furthermore, an *in silico* peptide screening pipeline was established to predict potential antioxidant peptides using deep-learning–based models, followed by experimental validation of the strain's antioxidant activities. By integrating genomic, computational, and experimental approaches, this study characterizes the antioxidant potential of *Cetobacterium ceti* strain MaLMAid0298 and provides a foundation for its further functional exploration.

## 2 Materials and methods

### 2.1 Strain isolation and cultivation

The strain MaLMAid0298 was isolated from the gill of *Sebastiscus marmoratus*, which was collected near Tongyeong, Korea, in July 2021. The strain was isolated under anaerobic conditions in Marine Broth (MB; BD Difco™, Michigan, USA) medium at 25 °C. MB was used for initial recovery because of its seawater-based composition. The culture was stored as a 20% glycerol stock (0.5 ml) at −80 °C.

To evaluate the optimal growth conditions of strain MaLMAid0298, growth assays were conducted under varying pH, temperature, and NaCl concentrations. Cultures were incubated anaerobically in 30 ml Hungate tubes, and OD_600_ was measured at 15–30 min intervals to calculate specific growth rates during the exponential phase. Temperature-dependent growth was assessed in Brain Heart Infusion (BHI; MB cell, Seoul, Korea) medium, a nutrient-rich medium used to support anaerobic growth during physiological characterization, at 20, 25, 32, 35.5, 40, and 45 °C. pH experiments were performed in the same medium at 35 °C, with the pH adjusted to 4–10 using 10 mm biological buffers. NaCl tolerance was tested in R2A (BD Difco™, Michigan, USA) medium supplemented with 0–2% NaCl (w/v) at 35 °C to allow controlled adjustment of salinity.

### 2.2 Whole-genome sequencing and assembly

Genomic DNA of *Cetobacterium ceti* strain MaLMAid0298 was extracted using the QIAamp DNA Mini Kit (Qiagen, Germany), and whole-genome sequencing was performed commercially (NICEM, Korea) using both the Illumina MiSeq platform (paired-end) and the Oxford Nanopore PromethION24 platform. Quality control of raw sequencing reads was conducted using FastQC v0.12.0 (Andrews, 2010). Adapter trimming and quality filtering were carried out using Trimmomatic v0.39 for Illumina data (Bolger et al., 2014). Raw Nanopore reads were basecalled using Dorado v7.8.3 with default adapter trimming. Hybrid genome assembly was performed with Unicycler v0.5.1 (Wick et al., 2017), and assembly quality was assessed using QUAST v5.3.0 (Gurevich et al., 2013). Circular genome maps were generated using Proksee (Grant et al., 2023).

### 2.3 Taxonomy and phylogeny analysis

The genomes of *Cetobacterium* strains and representative Fusobacteriaceae members used for genetic distance and comparative genomic analyses were retrieved from the NCBI RefSeq database and the Sequence Read Archive (SRA). Data obtained from the SRA (DRR354678, DRR354679, DRR354680) were processed into whole-genome assemblies following the same pipeline described above. Average Nucleotide Identity (ANI) was calculated with pyani v0.2.13.1 (Pritchard et al., 2016), and Average Amino Acid Identity (AAI) was assessed using CompareM v0.1.2 (https://github.com/dparks1134/CompareM). Digital DNA–DNA hybridization (dDDH) values were estimated using the Genome-to-Genome Distance Calculator (GGDC 3.0) provided by DSMZ.

Phylogenetic analysis was performed using the UBCG2 (Up-to-date Bacterial Core Genes 2) pipeline, which searches each genome for a pre-defined set of 81 conserved single-copy bacterial core genes (Kim et al., 2021). Comparative genomes were selected from available *Cetobacterium* genomes and representative Fusobacteriaceae genomes retrieved from NCBI RefSeq and SRA. The recovered UBCG2 core genes were aligned using MAFFT, and a maximum-likelihood tree was inferred using RAxML with 1,000 bootstrap replicates. The Gene Support Index (GSI) was calculated to evaluate the reliability of each node.

### 2.4 Gene prediction and functional annotation

Gene prediction and annotation were performed using Prokka v1.14.5 (Seemann, 2014). Functional annotation of predicted protein-coding genes was conducted using eggNOG-mapper v2.1.9 (Cantalapiedra et al., 2021) using the eggNOG v5.0 database with default parameters, which provided assignments of Clusters of Orthologous Groups (COG) categories, Gene Ontology (GO) terms, KEGG orthology (KO) numbers, and Pfam domain annotations (Huerta-Cepas et al., 2018).

Antioxidant-related genes were identified from RAST subsystem annotations, eggNOG-mapper functional assignments, KEGG orthology (KO) numbers, GO terms, Pfam domains, and Prokka product descriptions. Candidate genes were manually curated based on their functional relevance to oxidative stress defense and grouped into the five categories used in this study: core ROS scavenging systems, supporting redox systems, redox cofactor biosynthesis, protein quality control, and DNA protection and repair. Genes associated with peroxidases, thioredoxin/glutaredoxin systems, rubrerythrin/rubredoxin systems, NAD(P)H-dependent redox balance, Fe-S cluster and metal homeostasis, chaperones/proteases, and DNA repair were included. Genes were counted once based on coding sequences to avoid redundancy from overlapping annotations. Data visualization was performed using R v4.4.2 (R Core Team, 2024) with the ggplot2 package (Wickham, 2011).

### 2.5 *In silico* peptide generation and antioxidant activity prediction

Peptides ranging from 2 to 20 amino acids were generated from all predicted proteins of *Cetobacterium ceti* MaLMAid0298 using a sliding window approach (step size = 1), yielding 13,806,084 candidate sequences. Each peptide received a comprehensive antioxidant potential (AO) score based on amino acid composition and sequence context. The scoring considered residues frequently associated with antioxidant activity as well as aromatic motifs, histidine enrichment, terminal residue patterns, hydrophobicity, amphiphilicity, electronic stabilization descriptors, and metal chelation potential. Detailed scoring criteria and references are provided in Supplementary Table S1 (Sarmadi and Ismail, 2010; Power et al., 2013; Garrett et al., 2014; Zou et al., 2016; Shen et al., 2022).

Filtered peptides were processed with ESM-2 (esm2_t12_35M_UR50D model) to generate 480-dimensional structural embeddings (Lin et al., 2023). The smaller model variant was selected to enable direct peptide-level embedding at scale. Embeddings were reduced to 50 dimensions using UMAP (n_neighbors = 15, min_dist = 0.1, metric = “cosine”) for clustering, with parallel 2D projection for visualization (McInnes et al., 2018). *K*-means clustering (*k* = 1,000) identified structurally similar peptide families in the reduced embedding space. A weighted sampling strategy was employed to balance enrichment of high-quality candidates with structural diversity. Clusters were ranked by mean *z*-score, and representatives were selected as follows: 10 peptides from each of the top 100 clusters, three peptides from clusters ranked 101–500, and one peptide from clusters ranked 501–1000, yielding 2,700 representative peptides for experimental validation.

The 2,700 representative peptides were submitted to two independent machine learning-based antioxidant prediction tools for orthogonal validation. AnOxPePred predicted free radical scavenging and metal chelating activities, generating Scavenger and Chelator scores (0–1 scale) for each peptide (Olsen et al., 2020). Complementary predictions were obtained using AnOxPP, excluding 155 20-length-amino-acid peptides (Qin et al., 2023). Chelator scores were uniformly low across all peptides (maximum 0.36, median 0.20), indicating negligible metal chelating potential; subsequent analyses therefore focused on free radical scavenging activity. A threshold of 0.5 was applied to both Scavenger and AnOxPP scores for high-activity classification, based on median score distribution and consistency with literature-reported ranges. Peptides were categorized into consensus groups: “Both High” (Scavenger ≥0.5 and AnOxPP ≥0.5), “Single High” (one score ≥0.5), or “Both Low” (both scores <0.5). Pearson correlation coefficients were calculated to assess agreement between prediction methods. Cluster-level performance was evaluated by comparing mean prediction scores and consensus rates across tier groups. Length-dependent activity patterns were assessed by calculating mean scores with standard errors for each peptide length (2–20 aa). Top candidate peptides were ranked by combined score [(Scavenger + AnOxPP) / 2] within the “Both High” category, and the top 20 peptides were selected for further characterization. All statistical analyses and visualizations were performed in R v4.4.2 (R Core Team, 2024) using tidyverse (Wickham et al., 2019) for data manipulation and ggplot2 for visualization (Wickham, 2011).

### 2.6 *In vitro* assay preparation

The MaLMAid0298 strain was inoculated into 25 ml of 1/2 Marine Broth (1/2 MB) and cultured under anaerobic conditions at 25 °C for 5 days as the primary culture, following an in-house workflow used to prepare extracts from bacterial isolates under comparable conditions. Twenty-five milliliters of the primary culture were then transferred into 1 L of 1/2 MB and cultured under the same conditions for 2–3 days as the secondary culture. For large-scale cultivation, three 2.5 L Erlenmeyer flasks, each containing 1 L of 1/2 MB, were prepared. Twenty-five milliliters of the secondary culture were inoculated into each flask and cultured under anaerobic conditions at 25 °C for 5 days.

To the completed culture broth, sterile Amberlite^®^ XAD4, XAD7HP, and XAD16N resins (Sigma-Aldrich, St. Louis, MO, USA) were added at a 1:1:1 ratio (20 g per liter of culture broth). The resin-containing culture was agitated at 150 rpm at room temperature for 1 h to adsorb the metabolites of *Cetobacterium* sp. MaLMAid0298 onto the resins. The resins with adsorbed metabolites were separated using gauze, and the filtrate was discarded. Acetone was added to the recovered gauze and resins until fully submerged, and the mixture was agitated at 150 rpm at room temperature for 1 h to elute the adsorbed metabolites. The acetone eluate was collected and dried using a rotary vacuum evaporator to obtain the crude extract. The obtained extract was aliquoted into 20 mg portions and used for biological activity assays, including antioxidant, anti-inflammatory, antimicrobial, and anticancer activity analyses.

### 2.7 *In vitro* antioxidant and anti-inflammatory assays

DPPH (2,2-diphenyl-1-picrylhydrazyl) radical scavenging activity was measured using a 384-well plate. DPPH was dissolved in methanol at a concentration of 0.15 mM. Test samples and positive controls (ascorbic acid and α-tocopherol) were dispensed at 10 μl per well. Background absorbance was measured at 517 nm before adding 40 μl of DPPH solution. After adding DPPH solution, the plate was incubated at room temperature in the dark for 30 min, and absorbance was measured at 517 nm using a FlexStation 3 microplate reader. Radical scavenging activity was calculated using the following equation:


$$\begin{array}{l} \text{ Scavenging activity }\left(\text{\%}\right)=\\ \;\left\{1\;-\frac{\left(\text{O}{\text{D}}_{\text{sample},\text{ after}}\;-\text{ O}{\text{D}}_{\text{sample},\text{ before}}\right)}{\left(\text{O}{\text{D}}_{\text{control},\text{ after}}\;-\text{ O}{\text{D}}_{\text{control},\text{before}}\right)}\right\}\times \;100 \end{array}$$

For ABTS [2,2′-azino-bis(3-ethylbenzothiazoline-6-sulfonic acid)] radical scavenging activity measurement, 7 mm ABTS and 2.45 mm potassium persulfate were mixed at a 1:1 ratio and incubated at 4 °C in the dark for 12–16 h to generate ABTS radical cations. The reaction solution was diluted with ethanol to achieve an absorbance of 0.700 ± 0.100 at 734 nm. Test samples and positive controls (ascorbic acid and α-tocopherol) were dispensed at 10 μl per well in a 384-well plate, followed by the addition of 40 μl of the adjusted ABTS solution. After 15 min of incubation at room temperature in the dark, absorbance was measured at 734 nm using a FlexStation 3 microplate reader. Scavenging activity was calculated using the same equation as the DPPH assay.

- Intracellular ROS scavenging activity was evaluated using the DCFH-DA (Sigma-Aldrich, St. Louis, MO, USA) assay with Raw264.7 macrophages. Raw264.7 cells were cultured in DMEM medium supplemented with 10% FBS and 1% penicillin/streptomycin at 37 °C under 5% CO_2_. Cells were seeded in a 384-well plate at a density of 1 × 10^4^ cells/well and cultured for 24 h. Test samples and positive control (α-tocopherol) were added at various concentrations and incubated for 30 min, followed by treatment with lipopolysaccharide (LPS, Sigma-Aldrich) at a final concentration of 1 μg/ml for an additional 24 h. DCFH-DA was then added at a final concentration of 5μM and incubated for 30 min in the dark. Fluorescence was measured at 480/535 nm using an EnVision microplate reader. ROS scavenging activity was calculated using the following equation:

$$\begin{array}{l} \text{ROS scavenging }\left(\text{\%}\right)\;=\;\left\{1\;-\frac{\left({\text{F}}_{\text{sample}}-\;{\text{F}}_{\text{DMSO}}\right)}{\left({\text{F}}_{\text{LPS}}-\;{\text{F}}_{\text{DMSO}}\right)}\right\}\times \;100 \end{array}$$

Anti-inflammatory activity was evaluated by measuring nitric oxide (NO) production inhibition in LPS-stimulated Raw264.7 cells using the Griess reaction, following previously described approaches for preliminary anti-inflammatory activity evaluation (Ren and Chung, 2007; Inkanuwat et al., 2019). Cells were seeded in a 384-well plate at 1 × 104 cells/well and cultured for 24 h. Test samples and positive control (dexamethasone, 4 μg/ml) were added and incubated for 1 h, followed by treatment with 1 μg/ml LPS for 24 h. Culture supernatants were collected and reacted with Griess reagent according to the manufacturer's protocol. Absorbance was measured at 535 nm using an EnVision microplate reader. NO inhibition rate was calculated using the following equation:


$$\begin{array}{l} \text{NO inhibition }\left(\text{\%}\right)\;=\;\left\{1\;-\frac{\left(\text{O}{\text{D}}_{\text{sample}}-\text{ O}{\text{D}}_{\text{DMSO}}\right)}{\left(\text{O}{\text{D}}_{\text{LPS}}-\text{ O}{\text{D}}_{\text{DMSO}}\right)}\right\}\times \;100 \end{array}$$

## 3 Results

### 3.1 Morphological and physiological features of *Cetobacterium ceti* MaLMAid0298

The strain MaLMAid0298 was isolated from the gill of *Sebastiscus marmoratus* and exhibited phenotypic characteristics consistent with members of the genus *Cetobacterium*, including Gram-negative staining, non-motility, and growth only under anaerobic conditions. Physiological characterization revealed that strain MaLMAid0298 grew optimally at pH 7 (growth range pH 6–9), 40 °C (growth range 25–40 °C), and 2% NaCl (growth range 0.5–2%; Supplementary Figure S1). No growth was observed at pH 4, pH 10, 0% NaCl, or temperatures below 25 °C or above 40 °C.

### 3.2 Whole genome analysis of *Cetobacterium ceti* MaLMAid0298

The complete genome of MaLMAid0298 was assembled using a hybrid approach combining Illumina MiSeq short-read and Oxford Nanopore PromethION long-read sequencing data with Unicycler, achieving a sequencing depth of 731.3 × . The assembled genome comprises a closed circular chromosome (1,620,263 bp) with 11 circular plasmids, with a total genome size of 2,499,782 bp (Supplementary Figure S2). The GC content was 28.68%, consistent with the characteristically low GC content of the phylum Fusobacteriota. Genome annotation identified 2,322 coding sequences (CDSs), 99 tRNAs, 37 rRNAs, one tmRNA, and one repeat region. Detailed assembly and annotation statistics are provided in Table 1.

**Table 1:** Genomic characteristics of *C. ceti* strain MaLMAid0298.

Feature	Description
Strain name	MaLMAid0298
Genome size (bp)	2,499,782 (Chromosome: 1,620,263)
GC content (%)	28.68
No. of contigs	12
N50 (bp)	1,620,263
N90 (bp)	77,256
L50	1
L90	6
Gaps (N per 100 kb)	0
Sequencing coverage ( × )	731.3
Sequencing platforms	Illumina MiSeq/Oxford Nanopore PromethION
Assembly method	Unicycler (hybrid assembly)
Genome status	Complete genome
CDSs	2,322
tRNAs	99
rRNAs	37
tmRNA	1
Repeat regions	1

### 3.3 Phylogenetic position of MaLMAid0298 within the genus *Cetobacterium*

**Figure 1:**
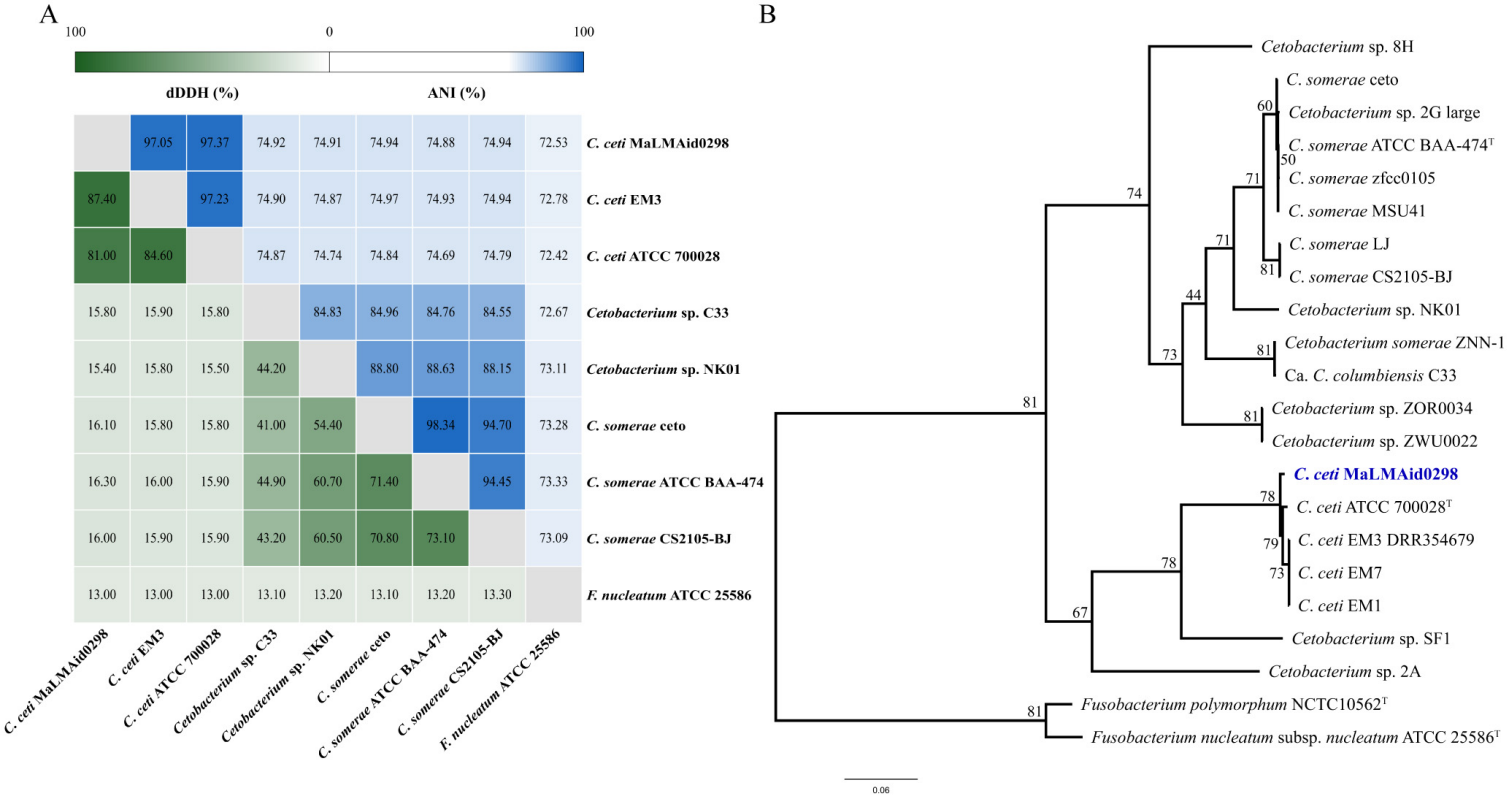
Genomic identification and phylogenetic placement of strain MaLMAid0298. **(A)** Pairwise average nucleotide identity (ANI, blue gradient) and digital DNA–DNA hybridization (dDDH, green gradient) values among strain MaLMAid0298 and representative *Cetobacterium* and *Fusobacterium* genomes. **(B)** Phylogenetic tree based on 81 bacterial core genes constructed using the UBCG2 pipeline. The tree was inferred using the maximum-likelihood (RAxML) method. Numbers at nodes indicate Gene Support Index (GSI) values based on 81 core genes. The study strain *C*. *ceti* MaLMAid0298 is highlighted in blue.

To determine the taxonomic placement of strain MaLMAid0298, average nucleotide identity (ANI) and digital DNA–DNA hybridization (dDDH) analyses were performed against representative *Cetobacterium* species and related taxa, with Fusobacterium nucleatum ATCC 25586 as an outgroup (Figure 1A). MaLMAid0298 exhibited 97.37% ANI and 81.00% dDDH similarity to the *C*. *ceti* type strain ATCC 700028^T^, values well above the established species delineation thresholds (95%−96% ANI; 70% dDDH), confirming its assignment to *C*. *ceti*. The highest dDDH value (87.40%) and ANI (97.23%) were observed with *C*. *ceti* strain EM3, indicating close genomic relatedness among *C*. *ceti* isolates. The strains of the *C*. *somerae* group displayed substantially lower similarity to MaLMAid0298 (dDDH 15.80–16.30%; ANI 74.88–74.94%).

A core-gene-based phylogenetic tree was constructed using 81 conserved bacterial genes via the UBCG2 pipeline to clarify the evolutionary relationship (Figure 1B). The strain MaLMAid0298 was placed within a highly supported *C*. *ceti* clade (Gene Support Index = 81), clustered with other strains, including the type strain ATCC 700028^T^. This clade was distinctly separated from the *C*. *somerae* lineage and other *Cetobacterium* species, further confirming its taxonomic identity as *C*. *ceti*.

### 3.4 Functional genome annotation related to antioxidant defense

**Figure 2:**
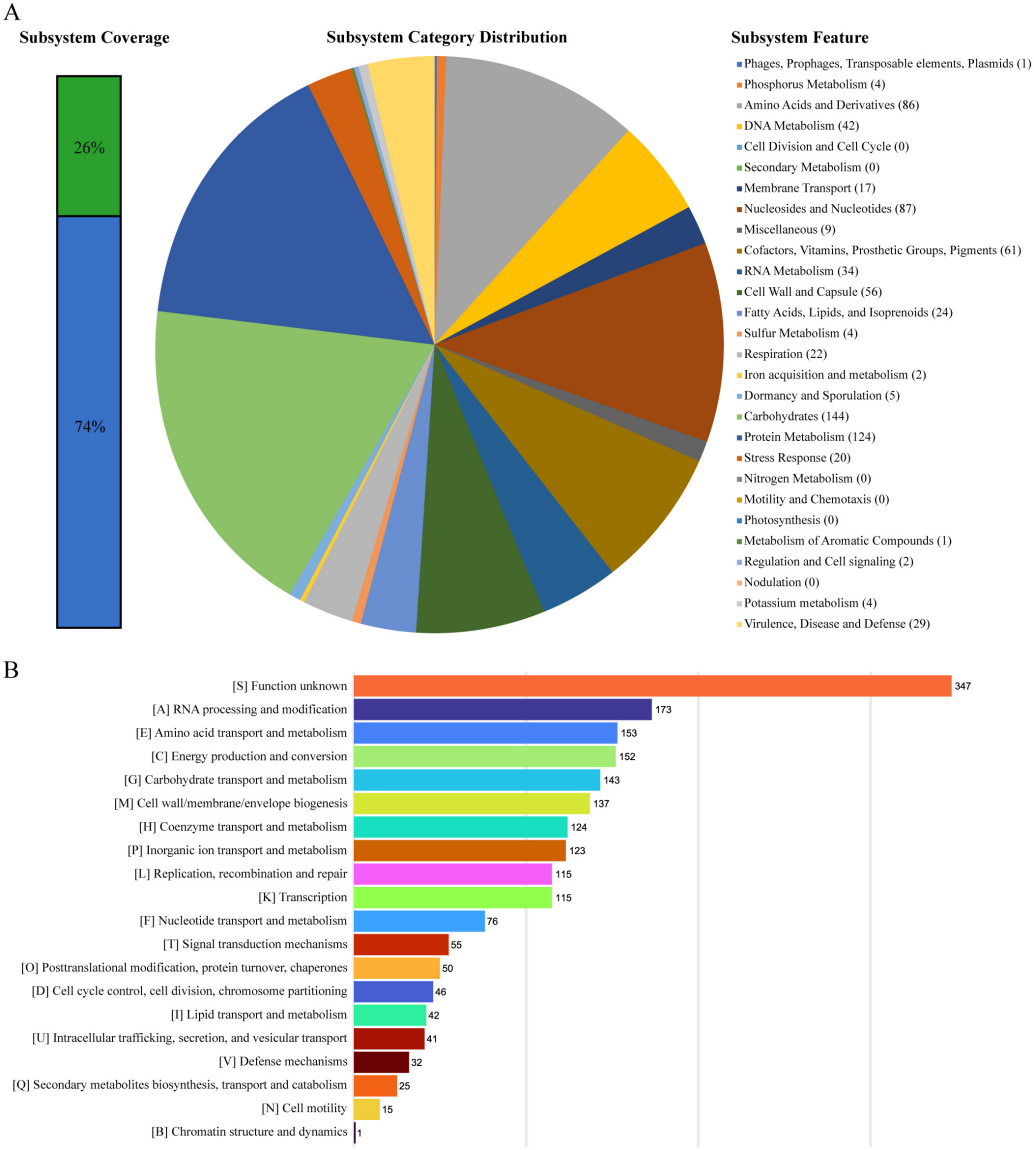
Functional annotation of the *C. ceti* MaLMAid0298 genome. **(A)** RAST subsystem-based functional categorization. The left panel shows subsystem coverage, with 26% of genes assigned to known subsystems. The right panel displays the distribution of genes across 27 subsystem categories, with Carbohydrates (144), Protein Metabolism (124), and Nucleosides and Nucleotides (87) being the most represented. **(B)** Distribution of genes across COG (Clusters of Orthologous Groups) functional categories. The most abundant categories were S (Function unknown, 347 genes), C (Energy production and conversion, 173), E (Amino acid transport and metabolism, 153), and J (Translation, ribosomal structure and biogenesis, 152).

To characterize the metabolic potential and stress response capabilities of *C. ceti* MaLMAid0298, functional annotation was performed using RAST and eggNOG-mapper. RAST annotation assigned 26% of the predicted genes to known subsystems, with the remaining 74% classified as hypothetical or uncharacterized proteins (Figure 2A). Among the annotated genes, the largest functional categories included Carbohydrates (144 genes), Protein Metabolism (124 genes), Amino Acids and Derivatives (86 genes), and Cofactors, Vitamins, Prosthetic Groups, and Pigments (61 genes). The Stress Response subsystem included 20 role assignments, corresponding to 14 coding sequences after duplicated features were merged. The 14 coding sequences were distributed across the following COG categories: Energy production and conversion (C; six genes), Inorganic ion transport and metabolism (P; two genes), Cell wall/membrane/envelope biogenesis (M; two genes), Signal transduction mechanisms (T; two genes), Transcription (*K*; one gene), and one gene assigned to both Transcription and Signal transduction mechanisms (*K*/T).

COG-based functional classification revealed a similar distribution pattern (Figure 2B). The most abundant categories were Energy production and conversion (C; 173 genes), Amino acid transport and metabolism (E; 153 genes), Translation, ribosomal structure and biogenesis (J; 152 genes), and Carbohydrate transport and metabolism (G; 143 genes). Categories directly relevant to oxidative stress defense included Posttranslational modification, protein turnover, and chaperones (O; 50 genes), Replication, recombination, and repair (L; 115 genes), and Coenzyme transport and metabolism (H; 124 genes). A substantial proportion of genes (347 genes) were assigned to the Function unknown category (S), reflecting the limited characterization of *Cetobacterium* genomes to date (Suzuki et al., 2022; Colorado Gómez et al., 2023; Qi et al., 2023).

Based on RAST subsystem annotation, eggNOG-mapper functional assignments, and manual curation of genes associated with ROS detoxification, thiol-redox balance, redox cofactor biosynthesis, protein quality control, and DNA repair, a total of 121 genes were identified as potentially involved in oxidative stress defense (Table 2). These genes were classified into five major functional categories: Core ROS scavenging systems (23 genes), Supporting systems (20 genes), Redox cofactor biosynthesis (39 genes), Protein quality control (17 genes), and DNA protection and repair (22 genes). MaLMAid0298 possessed a thioredoxin-centered antioxidant system (*trxA, trxB, grx*; 10 genes), which is associated with thiol-redox balance and bacterial oxidative stress tolerance (Imlay, 2013; Song et al., 2016). The genome also encoded the rubrerythrin/rubredoxin system (*rbr, rd, norV*; 6 genes), which is characteristic of anaerobic bacteria (Das et al., 2001). Peroxiredoxins (*ahpC, tpx*) and methionine sulfoxide reductases (*msrA, msrB*) may provide additional ROS detoxification capacity (Dubbs and Mongkolsuk, 2007; Grimaud et al., 2001). Supporting systems included NADPH-generating pentose phosphate pathway enzymes (*zwf* , *gnd*), the Rnf electron transfer complex (*rnfA, rnfB, rnfC, rnfD, rnfE, rnfG*), and Fe-S cluster biogenesis machinery (nifU, nifS, apbC), which may contribute to redox balance under anaerobic metabolism (Biegel et al., 2011; Imlay, 2013).

**Table 2:** Oxidative stress-related genes identified in the *C. ceti* MaLMAid0298 genome.

Functional category	Subcategory	Representative genes	Function
Core ROS scavenging systems (23)	Thioredoxin system (10)	*trxA, trxB, grx*	Thiol-disulfide oxidoreductase; maintains cellular redox balance
	Peroxidase (2)	*ahpC, tpx*	Thiol peroxidase; H_2_O_2_ and organic hydroperoxide reduction
	Rubrerythrin/Rubredoxin (6)	*rbr, rd, norV*	anaerobic ROS/nitrosative stress detoxification
	Methionine sulfoxide reductase (3)	*msrA, msrB*	Repairs oxidized methionine residues in proteins
	ROS metabolism (2)	*noxE*	NADH oxidase; oxygen tolerance protein
Supporting systems (20)	Redox regulation (2)	*Rex*	NAD^+^/NADH-responsive transcriptional regulator
	NADPH generation (PPP) (2)	*zwf, gnd*	Pentose phosphate pathway enzymes; NADPH supply
	Electron transfer (Fd/Fld) (4)	*fldA, isiB*	Flavodoxin; low-potential electron carriers
	Electron transfer (Rnf complex) (6)	*rnfA/B/C/D/E/G*	Membrane ferredoxin:NAD^+^ oxidoreductase
	Fe-S cluster/Iron homeostasis (6)	*nifU, nifS, feoB*	Fe-S cluster assembly; iron uptake
Redox cofactor biosynthesis (39)	NAD/NADP biosynthesis (6)	*nadD, nadE, nadK*	NAD^+^/NADP^+^ *de novo* and salvage pathways
	Heme biosynthesis (6)	*hemA, hemB, hemL*	Tetrapyrrole biosynthesis; rubrerythrin cofactor
	Vitamin B2 biosynthesis (5)	*ribD, ribE, ribF*	Riboflavin/FAD/FMN biosynthesis
	Vitamin B12 biosynthesis (22)	*cbiA/C/D/E/G/M/N/T/X, cobA/D/I/J/K/M/O/Q/S/T/U*	Anaerobic cobalamin biosynthesis pathway
Protein quality control (17)	Chaperone/Heat shock (11)	*dnaK, groEL, clpB*	Molecular chaperones; protein folding and disaggregation
	Chaperone/Heat shock (5)	*clpXP, lon, hslUV*	ATP-dependent proteases; damaged protein degradation
	Chaperone/Heat shock (1)	*hslO*	Redox-regulated chaperone (Hsp33)
DNA protection and repair (22)	DNA protection (2)	*dps, ybaB*	DNA-binding ferritin; nucleoid organization
	DNA repair (BER) (5)	*nth, nfo, ogt*	Base excision repair; oxidized base removal
	DNA repair (HR) (8)	*recA, recG, ruvA/B/C*	Homologous recombination; DSB repair
	DNA repair (NER) (5)	*uvrA/B/C, mfd*	Nucleotide excision repair; bulky lesion removal
	DNA repair (MMR) (2)	*mutS, mutL*	Mismatch repair; replication fidelity

Numbers in parentheses indicate the number of genes included in each functional category or subcategory.

Redox cofactor biosynthesis represented the largest category, with a complete cobalamin biosynthesis pathway (vitamin B_12_, 22 genes), riboflavin/FMN/FAD metabolism-related genes (vitamin B_2_; five genes), and heme biosynthesis-related genes (six genes). Protein quality control systems (DnaK-DnaJ-GrpE, GroEL-GroES, ATP-dependent proteases) and DNA repair mechanisms (BER, HR, NER, MMR) may further contribute to protection against oxidative damage to macromolecules (Imlay, 2013). KEGG module-based reconstruction supported a complete anaerobic cobalamin-related biosynthesis pathway in MaLMAid0298, including M00846, M00924, and M00122 (Supplementary Figure S3). Riboflavin metabolism-related genes, including *ribBA, ribD, ribE, ribH*, and *ribF*, were also identified and mapped to riboflavin metabolism (Supplementary Figure S3). However, the KEGG riboflavin biosynthesis module M00125 was not recovered as complete because a phosphatase-related step was not assigned.

### 3.5 Peptide-based antioxidant prediction and candidate selection

**Figure 3:**
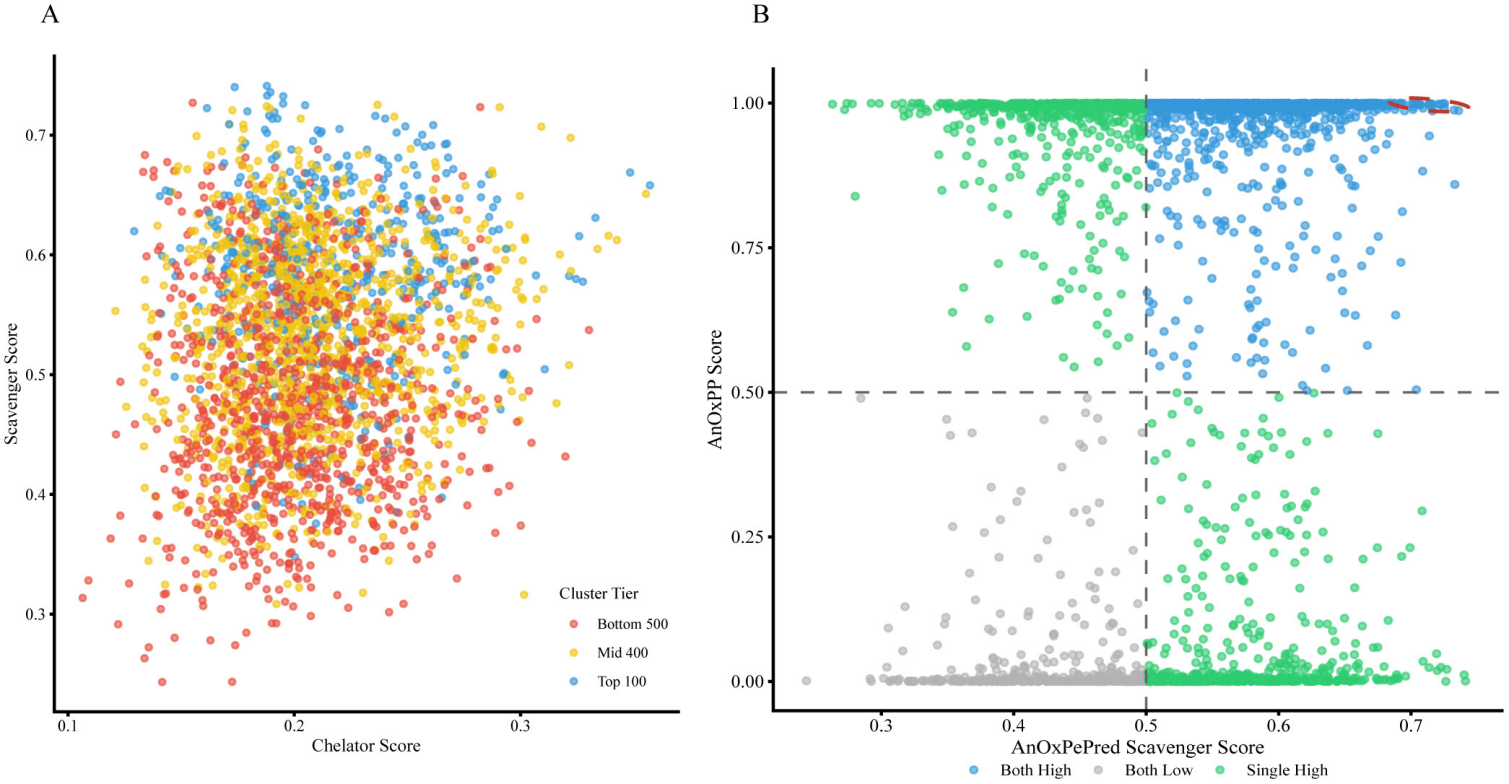
Machine learning-based antioxidant activity predictions for candidate peptides. **(A)** Scatter plot of AnOxPePred Scavenger vs. Chelator scores for 2,700 representative peptides, colored by cluster tier (Top 100, Mid 400, Bottom 500). Chelator scores were uniformly low (maximum 0.36), while Scavenger scores showed greater variation across tiers. **(B)** Consensus classification based on AnOxPePred Scavenger and AnOxPP scores. Dashed lines indicate the 0.5 threshold for high-activity classification. Peptides were categorized as “Both High” (blue), “Single High” (green), or “Both Low” (gray). The red dotted circle indicates the top 20 candidate peptides selected for further characterization.

To validate the rule-based antioxidant scoring system, 2,700 representative peptides were evaluated using two independent machine learning prediction tools. AnOxPePred analysis revealed distinct distributions between Scavenger and Chelator activities (Figure 3A). Scavenger scores ranged from 0.28 to 0.74 (median 0.52), while Chelator scores were consistently low across all peptides (range 0.10–0.36, median 0.20), indicating that the selected peptide candidates pre-dominantly exhibit free radical scavenging rather than metal chelating potential. Peptides from Top 100 clusters showed higher Scavenger scores (mean 0.583) compared to Bottom 500 clusters (mean 0.477), confirming the effectiveness of *z*-score-based cluster ranking. AnOxPP analysis was successfully completed for 2,545 peptides, with 155 peptides (5.7%) excluded due to the tool's 19 amino acid length limitation. Among the analyzed peptides, 1,576 (61.9%) were classified as antioxidant peptides.

Consensus classification between the two prediction tools was visualized by plotting AnOxPePred Scavenger scores against AnOxPP scores (Figure 3B). A threshold of 0.5 was applied to both axes, dividing peptides into four quadrants. Peptides in the upper-right quadrant (Scavenger ≥0.5 and AnOxPP ≥0.5) were classified as “Both High,” representing 997 peptides (39.2%) with robust antioxidant potential validated by both independent algorithms. Peptides showing high scores in only one method were classified as “Single High” (535 Scavenger-only and 579 AnOxPP-only), while 434 peptides below threshold in both were classified as “Both Low”. Tier-level analysis revealed that peptides from Top 100 clusters demonstrated the highest consensus rate (45.2% Both High), compared to Mid 400 clusters (43.0%) and Bottom 500 clusters (25.2%), further supporting the validity of the rule-based prioritization strategy.

Length-dependent activity patterns showed divergent trends between prediction methods (Supplementary Figure S4). AnOxPP scores were highest for short peptides (2–4 aa) and decreased with increasing length, while AnOxPePred Scavenger scores showed relatively stable values across the length range. The rule-based AO *Z*-scores displayed a similar decreasing trend with length, reflecting the *z*-score normalization applied within each length group. Chelator scores remained uniformly low regardless of peptide length.

The top 20 candidate peptides were selected from the “Both High” category based on combined scores [(Scavenger + AnOxPP) / 2] (Figure 3B, Table 3). These candidates were enriched in aromatic residues, particularly tyrosine repeats (e.g., YYYGH, FYYYY) and histidine-tryptophan motifs (e.g., FFHWGFHPWGIY, LGLHWYHFKH). The majority of top candidates originated from Top 100 (55%) and Mid 400 (45%) clusters, with none from Bottom 500 clusters. The highest-scoring peptide, YYYGH, exhibited a Scavenger score of 0.72 and an AnOxPP score of 0.997. Complete prediction results for all 2,700 representative peptides, including sequence information, cluster assignments, tier classification, and prediction scores from both tools, are provided in Supplementary Table S2.

**Table 3:** Top 20 predicted antioxidant peptide candidates from *C. ceti* MaLMAid0298.

Sequence	length	AO_score	Cluster tier	Chelator	Scavenger	AnOxPP
YLGFTFLPYYYFIFLFF	17	28.5	Mid 400	0.23676	0.72532	0.999
FYYYY	5	17.5	Mid 400	0.18786	0.72416	0.9992
YVLFNYHYYFSH	12	27.9	Top 100	0.18948	0.73587	0.9869
VLFNYHYYFSH	11	26	Top 100	0.19513	0.73251	0.988
YYYGH	5	16.7	Mid 400	0.18935	0.72213	0.9974
FLGYYYGH	8	21.6	Mid 400	0.17268	0.72074	0.9984
YYYH	4	17.2	Top 100	0.20479	0.725	0.992
YLSFTFLPYYYFIFLFF	17	28.5	Mid 400	0.2442	0.71544	0.999
LGLHWYHFKH	10	26.9	Top 100	0.23581	0.71609	0.9971
FTFLPYYYFIFLF	13	24.2	Mid 400	0.20287	0.7138	0.9992
GLHWYHFKH	9	25.9	Top 100	0.24017	0.71384	0.9964
YHYY	4	15.7	Top 100	0.21998	0.70516	0.9992
FTFLPYYYFIFLFF	14	26.6	Mid 400	0.17769	0.70519	0.999
FFHWGFHPWGIYSF	14	32.2	Top 100	0.24908	0.70729	0.9968
FHELTHLIYPHHQKEFYEF	19	30	Mid 400	0.30914	0.70711	0.9969
YHYYMY	6	19.1	Top 100	0.18702	0.70452	0.9982
LFNYHYYFSH	10	26	Top 100	0.18892	0.7162	0.9863
HYYY	4	16.2	Top 100	0.2121	0.70302	0.9991
FFHWGFHPWGIY	12	30.8	Top 100	0.24522	0.70217	0.9996
FTFLPYYYFIFLFFI	15	26.6	Mid 400	0.17128	0.70906	0.991

### 3.6 Experimental validation of antioxidant and anti-inflammatory activities

**Figure 4:**
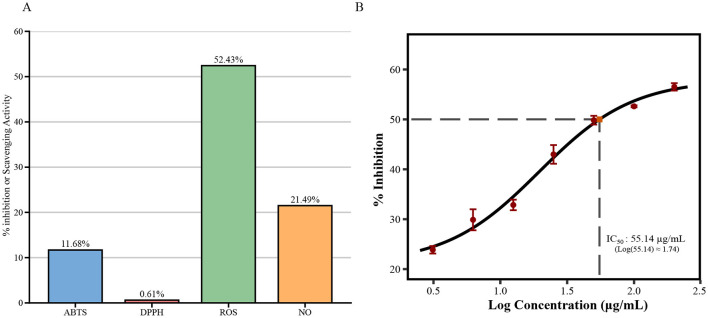
*In vitro* antioxidant and anti-inflammatory activities of *C. ceti* MaLMAid0298. **(A)** Comparative evaluation of the antioxidant (ABTS, DPPH, ROS) and anti-inflammatory (NO) activities at 50 μg/ml. Activities are expressed as mean percentage inhibition or scavenging values (%). **(B)** Dose-dependent ROS scavenging activity of strain MaLMAid0298. The percentage inhibition of intracellular ROS was plotted against log-transformed concentrations of the crude extract. The half-maximal inhibitory concentration (IC_50_) was determined to be 55.14 μg/ml (log_10_ 55.14 ≈ 1.74), as indicated by the intersection of the gray dashed lines and the orange square.

To evaluate the bioactive potential of *C*. *ceti* MaLMAid0298, crude extracts were prepared and subjected to a series of antioxidant and anti-inflammatory assays. The antioxidant capacity was assessed using four complementary methods: DPPH radical scavenging, ABTS radical scavenging, cellular ROS inhibition (DCFH-DA assay), and nitric oxide (NO) inhibition (Griess assay; Figure 4A).

Among the cell-free radical scavenging assays, ABTS radical scavenging activity was 11.68%, whereas DPPH radical scavenging activity was negligible (0.61%), indicating limited direct radical scavenging activity under the tested conditions. In the cell-based DCFH-DA assay, the extract reduced DCF-associated fluorescence by 52.43% at 50 μg/ml, whereas the α-tocopherol positive control showed 70.68% inhibition under the same assay conditions. This cellular response was therefore lower than that of α-tocopherol but more pronounced than the direct radical scavenging activities observed in the DPPH and ABTS assays. The extract also reduced NO production by 21.49%, compared with 60.01% for the positive control. Because DCF-based fluorescence assays can be affected by dye oxidation, background fluorescence, and sample-dependent interactions, the cellular assay result was interpreted as a preliminary redox-associated readout rather than direct quantitative evidence of intracellular ROS elimination (Karlsson et al., 2010; Korshunov and Imlay, 2025).

Dose-response analysis of cellular ROS inhibition yielded an IC_50_ value of 55.14 μg/ml, confirming concentration-dependent activity (Figure 4B). The pronounced discrepancy between chemical and cellular antioxidant activities suggests that the bioactive components in the extract may function through interactions with intracellular antioxidant defense systems rather than direct radical scavenging mechanisms.

## 4 Discussion

In this study, we characterized the genome of MaLMAid0298 together with preliminary *in vitro* functional assays. Phylogenomic analyses confirmed that MaLMAid0298 belongs to the *Cetobacterium ceti* clade. The genome of *C*. *ceti* MaLMAid0298 was found to have antioxidant potential at both gene and peptide levels. Such an ability may be required to survive in oxygen-variable environments, such as fish gills or intestines, as a host-adapted anaerobe. The genome exhibited low GC content (28.68%) with a small chromosome size (1.6 Mbp), as reported for other Fusobacteriota species (Brewer and Wagner, 2021). In addition, the abundance of plasmids may be associated with adaptation to the host-associated environment (Wein and Dagan, 2020; Rodríguez-Beltrán et al., 2021).

In the fish gill environment, persistence of anaerobic microorganisms depends on adaptation to fluctuating conditions. Oxygen availability, in particular, is not uniform. At the gill surface, microgradients can form within the boundary layer of mucus (Shephard, 1992). These gradients may shift rapidly as ventilation patterns and water flow alter diffusion distances and effective surface area (Wood and Eom, 2021). Even niches that are generally considered low in oxygen are not necessarily free from oxidative stress, because host immune activity can generate ROS locally (Biller and Takahashi, 2018). The genomic features of MaLMAid0298 are consistent with potential tolerance to intermittent oxidative exposure, but this hypothesis requires validation under oxidative-stress conditions.

Previous *C. ceti* isolates have been reported from marine mammals, including a harbor porpoise intestinal-content isolate and a minke whale mouth-lesion isolate, and more recent strains were isolated from fecal samples of captive common bottlenose dolphins (Foster et al., 1995; Suzuki et al., 2022). In contrast, MaLMAid0298 was isolated from the gill of the marine fish *Sebastiscus marmoratus*. Because publicly available *C. ceti* genome data remain limited, direct comparison of host-associated physiology and antioxidant-related traits is currently restricted. Further comparative studies using *C. ceti* isolates from different host sources will be required to determine whether cobalamin biosynthesis, redox-related systems, or stress-response features vary according to host habitat.

As expected for many strict anaerobes, MaLMAid0298 lacks catalase and superoxide dismutase, enzymes that are commonly central to oxidative stress defense in aerobic bacteria (Imlay, 2013). Instead, MaLMAid0298 appears to rely on oxygen-independent antioxidant defenses. Among these, the thioredoxin-centered thiol-redox network (*trxA, trxB, grx*) plays a central role in maintaining intracellular redox homeostasis and limiting oxidative stress, including host-derived ROS (Song et al., 2016). Additional protection is provided by the rubrerythrin/rubredoxin system (*rbr, rd*) (Das et al., 2001) and a nitric oxide reductase (*norV*) implicated in anaerobic NO detoxification and nitrosative-stress tolerance (Shimizu et al., 2015; Liu et al., 2019). The genome also encodes peroxide-detoxifying enzymes, including peroxiredoxins (*ahpC, tpx*) (Dubbs and Mongkolsuk, 2007) and methionine sulfoxide reductases (*msrA, msrB*) that repair oxidized proteins (Grimaud et al., 2001). The presence of the Rnf electron transfer complex may further contribute to maintaining redox balance under anaerobic metabolism (Biegel et al., 2011). These features support the hypothesis that MaLMAid0298 may rely on thiol-based redox systems, although this requires further experimental validation.

Consistent with previous reports identifying *Cetobacterium* species as cobalamin (vitamin B1_2_) producers in fish intestinal microbiomes (Tsuchiya et al., 2008; Suzuki et al., 2022; Colorado Gómez et al., 2023; Qi et al., 2023; Zhu et al., 2025), MaLMAid0298 has a complete anaerobic cobalamin biosynthesis pathway. This oxygen-independent route is well-suited to the microaerophilic gut environment and may contribute to host nutrition. Riboflavin (vitamin B_2_) and NAD^+^/NADP^+^ biosynthesis pathways provide essential cofactors for the thioredoxin and rubrerythrin systems described above. KEGG reconstruction indicated that the riboflavin-related genes mapped to riboflavin metabolism (map00740), but the riboflavin biosynthesis module M00125 was not recovered as complete because a phosphatase-related step was not assigned. Vitamin B_12_ and riboflavin have been associated with antioxidant-related functions, suggesting that these pathways may be relevant to the redox potential of MaLMAid0298 (van de Lagemaat et al., 2019; Olfat et al., 2022). Direct quantification of vitamin production and its contribution to antioxidant activity should be addressed in future studies.

To explore antioxidant potential beyond dedicated redox enzymes, we applied an *in silico* peptide screening strategy using the MaLMAid0298 genome sequence. This approach enabled an unbiased search for short peptide fragments derived from diverse proteins. Predictions from two independent models indicated enrichment of free radical scavenging potential among selected candidates, whereas predicted metal-chelating activity was comparatively low. High-scoring peptides frequently contained aromatic residues (Tyr, Trp, and Phe) and histidine-containing motifs, consistent with previously reported sequence features associated with radical scavenging activity (Power et al., 2013; Garrett et al., 2014; Zou et al., 2016). These candidate peptides were distributed across diverse protein backgrounds and were not confined to dedicated redox enzymes, suggesting that antioxidant potential in MaLMAid0298 may not be limited to known antioxidant proteins. Deep learning models that incorporate structural information may identify candidates that are not readily captured by traditional homology-based approaches.

The crude extract of MaLMAid0298 reduced DCF-associated fluorescence in the cell-based assay, with an inhibition rate of 52.43% (IC_50_ = 55.14 μg/ml). In contrast, radical scavenging activity in chemical assays was minimal, with DPPH and ABTS scavenging of 0.61% and 11.68%, respectively. This discrepancy suggests that the extract does not primarily function as a direct radical scavenger in cell-free systems (Tirzitis and Bartosz, 2010). Instead, the bioactive components may exert indirect effects in cells (e.g., by influencing cellular redox homeostasis, antioxidant response pathways, or inflammatory signaling) (Nwachukwu et al., 2021). The underlying mechanism remains unclear, as the assays were conducted using crude extracts and a single cell line.

Functional assays were performed using crude extracts, which limits direct attribution of activity to specific compounds or peptides. The genome sequence and functional annotations of *C*. *ceti* MaLMAid0298 provide a genetic basis for its antioxidant features. The genomic and preliminary functional features of MaLMAid0298 suggest that fish-associated *C. ceti* strains may be worth evaluating as probiotic candidates in future studies. In particular, cobalamin biosynthetic capacity and redox-related genomic features may be relevant to host nutrition and stress physiology. However, probiotic application would require further validation, including safety assessment, host colonization ability, stability under rearing conditions, and *in vivo* trials evaluating growth, immune response, oxidative stress markers, and pathogen resistance. Together, these findings provide an integrated view of the antioxidant capacity of MaLMAid0298 and support its further exploration for functional applications.

## Data Availability

The datasets presented in this study can be found in online repositories. The names of the repository/repositories and accession number(s) can be found below: NCBI GenBank under BioProject PRJNA1367294, BioSample SAMN53336707, and accession number JBSLQM000000000.

## Supplementary material

SUPPLEMENTARY FIGURE S1 Effect of pH, NaCl Concentration, and Temperature on the Specific Growth Rate of Strain MaLMAid0298. **(A)** Effect of pH (4–10), **(B)** Effect of NaCl concentration (0%−2%), and **(C)** Effect of temperature (20–45 °C).

SUPPLEMENTARY FIGURE S2 Complete genome map of *Cetobacterium ceti* MaLMAid0298. The circular map consists of one chromosome (top) and 11 plasmids (bottom). Chromosome size is 1,620,263 bp, and the plasmid sizes range from 2,501 bp to 182,596 bp. From the outermost to innermost rings, the map displays coding sequences (CDSs) on the forward and reverse strands, rRNAs, tRNAs, GC content, and GC skew.

SUPPLEMENTARY FIGURE S3 KEGG-based reconstruction of cobalamin and riboflavin-related pathways in *C. ceti* MaLMAid0298. **(A)** KEGG module reconstruction supported a complete anaerobic cobalamin-related biosynthesis pathway, including M00846, M00924, and M00122. **(B)** Riboflavin-related genes mapped to riboflavin metabolism, but module M00125 was not recovered as complete because a phosphatase-related step was not assigned.

SUPPLEMENTARY FIGURE S4 Length-dependent antioxidant prediction scores. Mean scores (±SE) for peptides of 2–20 amino acids. Short peptides (2–5 aa) showed higher AnOxPP and AO Z-scores, while Scavenger scores remained stable and Chelator scores were uniformly low across all lengths.
