# Supplementary material for: Antioxidant potential and genomic adaptation of Cetobacterium ceti MaLMAid0298 from the gills of Sebastiscus marmoratus
Source: Front Microbiol. 2026 Jun 25;17:1815687. doi: 10.3389/fmicb.2026.1815687 (PMC13346224; doi:10.3389/fmicb.2026.1815687)
Supplement: Supplementary file 1 [file Table_1.docx]

**Supplementary Table S1.** Scoring criteria for predicting antioxidant peptide activity based on structural and compositional features.

| **Category** | **Scoring Feature** | **Criteria** | **Score** | **Rule** | **Reference(s)** |
| --- | --- | --- | --- | --- | --- |
| Antioxidant residues | Core residues | Y, W, H, C, M present (each) | +1 | ^a^ | Power et al., 2013; Zou et al., 2016 |
|  | Phenylalanine | F present | +0.5 |  | Sarmadi & Ismail, 2010 |
| Electronic stabilization | sp²-hybridized carbons | Per sp² carbon in peptide | +0.4 |  | Garrett et al., 2014 |
|  | E/C ratio | Mean E/C ≥ 2.3 | +2 | ^b^ | Garrett et al., 2014 |
|  |  | Mean E/C 2.0–2.3 | +1 | ^b^ | Garrett et al., 2014 |
| Metal chelation | Histidine enrichment | His ratio ≥ 0.10 | +2 |  | Zou et al., 2016 |
|  | C-terminal His | His at C-terminus | +1 | ^d^ | Zou et al., 2016 |
| Sequence context | Aromatic adjacency | Adjacent W/Y/H/F pairs | +1 | ^c^ | Garrett et al., 2014; Power et al., 2013 |
|  | Proline β-turn | Pro at i±1 from aromatic | +0.5 |  | Zou et al., 2016; Shen et al., 2022 |
|  | N-terminal hydrophobic | N-term = L/I/V/F/W/P | +1 |  | Garrett et al., 2014; Power et al., 2013 |
|  | C-terminal electronics | C-term = W/Y/C/H/M | +1 | ^a^ | Power et al., 2013; Zou et al., 2016 |
|  | Cys-Trp proximity | Cys and Trp ≤3 residues | +0.5 | ^e^ | Garrett et al., 2014; Power et al., 2013 |
| Motifs | Known AO dipeptides | YH, HY, YY, YW, WY, YC, CY, WH, HW, WW, WC, CW, VH, HV, LH, HL, PH, HP, AH, HA, IH, HI, EC, CE, CC, VY, YV, LY, YL, IY, YI, VW, WV, LW, WL, IW, WI, PY, YP, PW, WP, PF, FP | +0.5/motif | ^c^ | Sarmadi & Ismail, 2010; Shen et al., 2022 |
|  | Known AO tripeptides | PYF, PWF, PYY, PWW, PYW, PWY, YYY, WWW, YWY, WYW, LYH, VYH, IYH | +1.0/motif | ^c^ | Sarmadi & Ismail, 2010; Shen et al., 2022 |
| Physicochemical | Hydrophobicity ratio | Ratio 0.30–0.60 | +1 |  | Sarmadi & Ismail, 2010; Power et al., 2013; Shen et al., 2022 |
|  | Amphiphilicity index | Index 0.30–0.55 | +1 |  | Sarmadi & Ismail, 2010; Zou et al., 2016 |

a. Applied once per residue type. Positional bonuses (N-/C-terminal) are cumulative as they reflect distinct mechanistic contributions.

b. E/C ratio ranges are mutually exclusive; apply the higher score only.

c. Aromatic adjacency and motif bonuses are mutually exclusive for the same residue pair to prevent double-counting.

d. C-terminal His receives both metal chelation and C-terminal electronics bonuses, reflecting enhanced binding capacity at this position (Zou et al., 2016).

e. Cys-Trp proximity bonus (+0.5) is additive to individual residue scores, reduced from +1.5 to avoid over-weighting synergy effects.
