# Supplementary figures and images for: Antioxidant potential and genomic adaptation of Cetobacterium ceti MaLMAid0298 from the gills of Sebastiscus marmoratus
Source: Front Microbiol. 2026 Jun 25;17:1815687. doi: 10.3389/fmicb.2026.1815687 (PMC13346224; doi:10.3389/fmicb.2026.1815687)

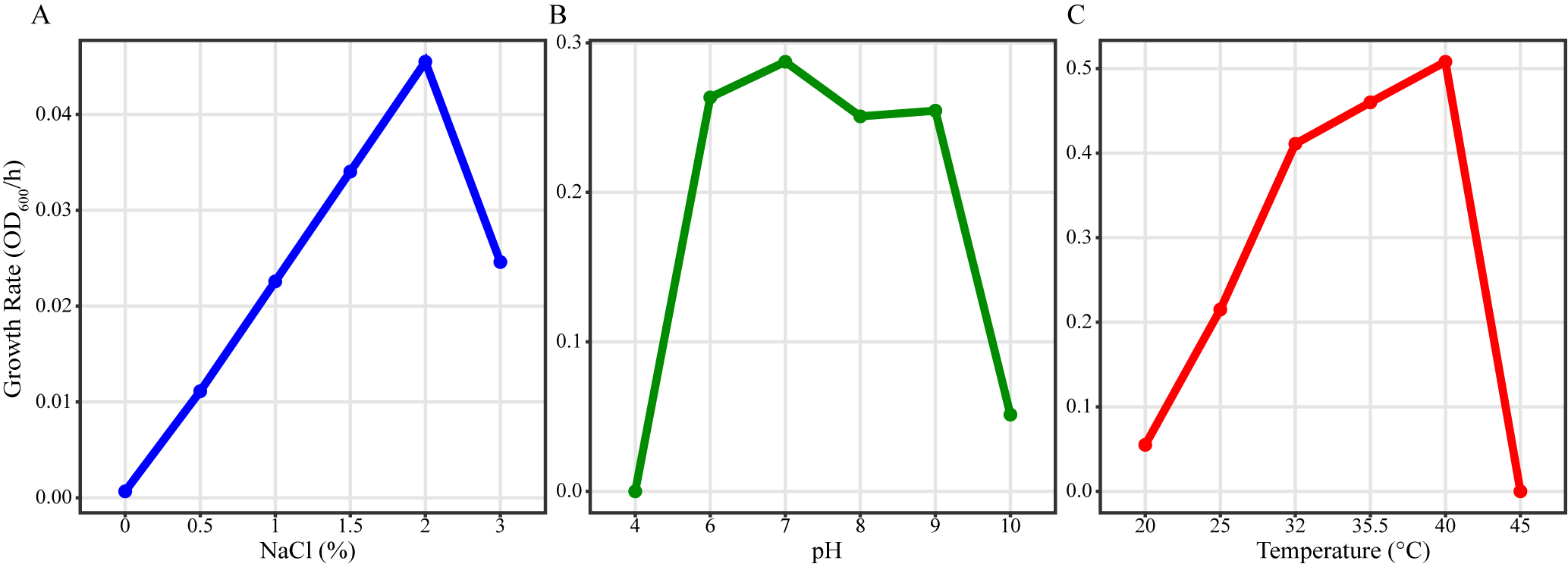

Supplement: SUPPLEMENTARY FIGURE S1 — Effect of pH, NaCl Concentration, and Temperature on the Specific Growth Rate of Strain MaLMAid0298. (A) Effect of pH (4–10), (B) Effect of NaCl concentration (0%−2%), and (C) Effect of temperature (20–45 °C). [file Image_1.TIF]

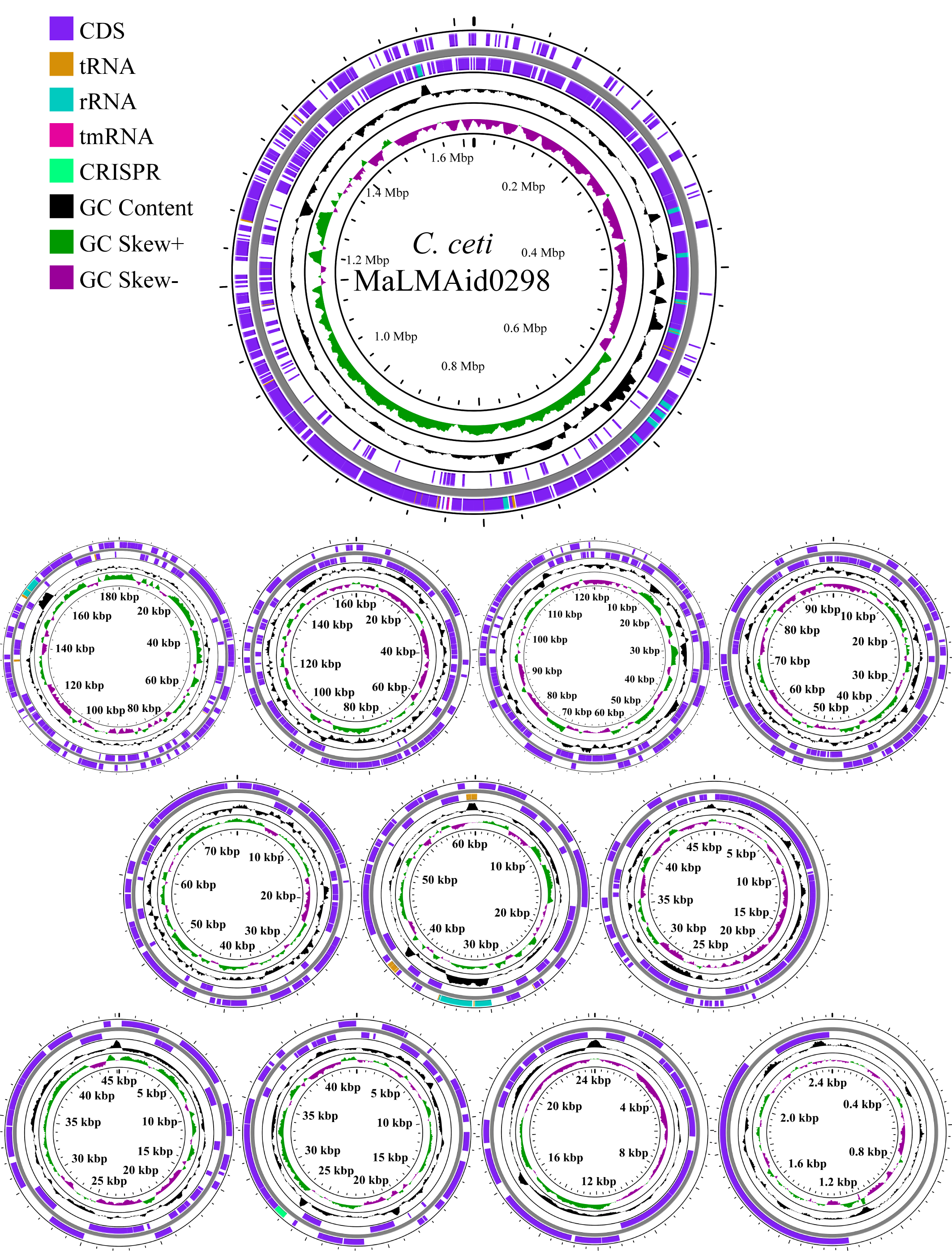

Supplement: SUPPLEMENTARY FIGURE S2 — Complete genome map of Cetobacterium ceti MaLMAid0298. The circular map consists of one chromosome (top) and 11 plasmids (bottom). Chromosome size is 1,620,263 bp, and the plasmid sizes range from 2,501 bp to 182,596 bp. From the outermost to innermost rings, the map displays coding sequences (CDSs) on the forward and reverse strands, rRNAs, tRNAs, GC content, and GC skew. [file Image_2.TIF]

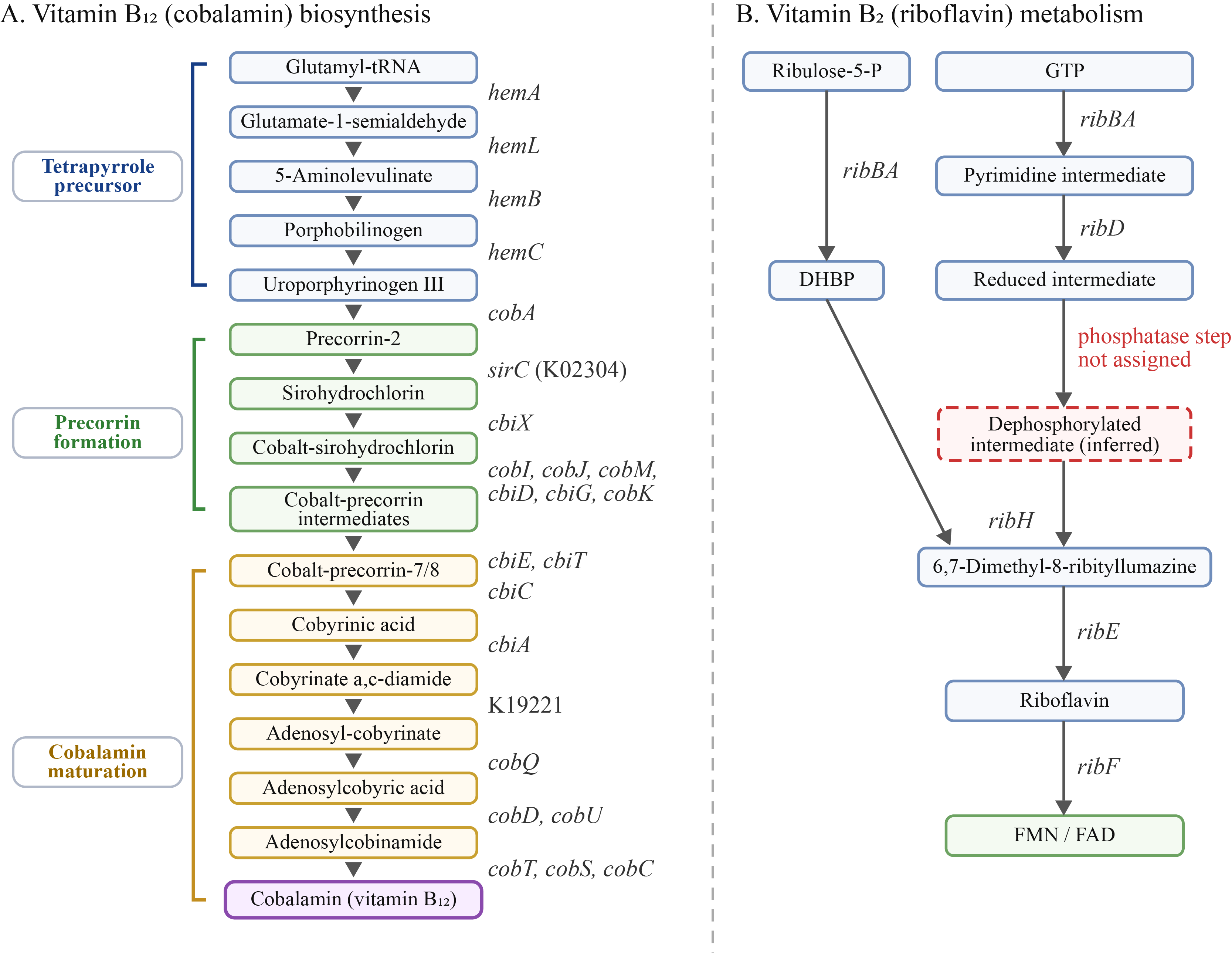

Supplement: SUPPLEMENTARY FIGURE S3 — KEGG-based reconstruction of cobalamin and riboflavin-related pathways in C. ceti MaLMAid0298. (A) KEGG module reconstruction supported a complete anaerobic cobalamin-related biosynthesis pathway, including M00846, M00924, and M00122. (B) Riboflavin-related genes mapped to riboflavin metabolism, but module M00125 was not recovered as complete because a phosphatase-related step was not assigned. [file Image_3.TIF]

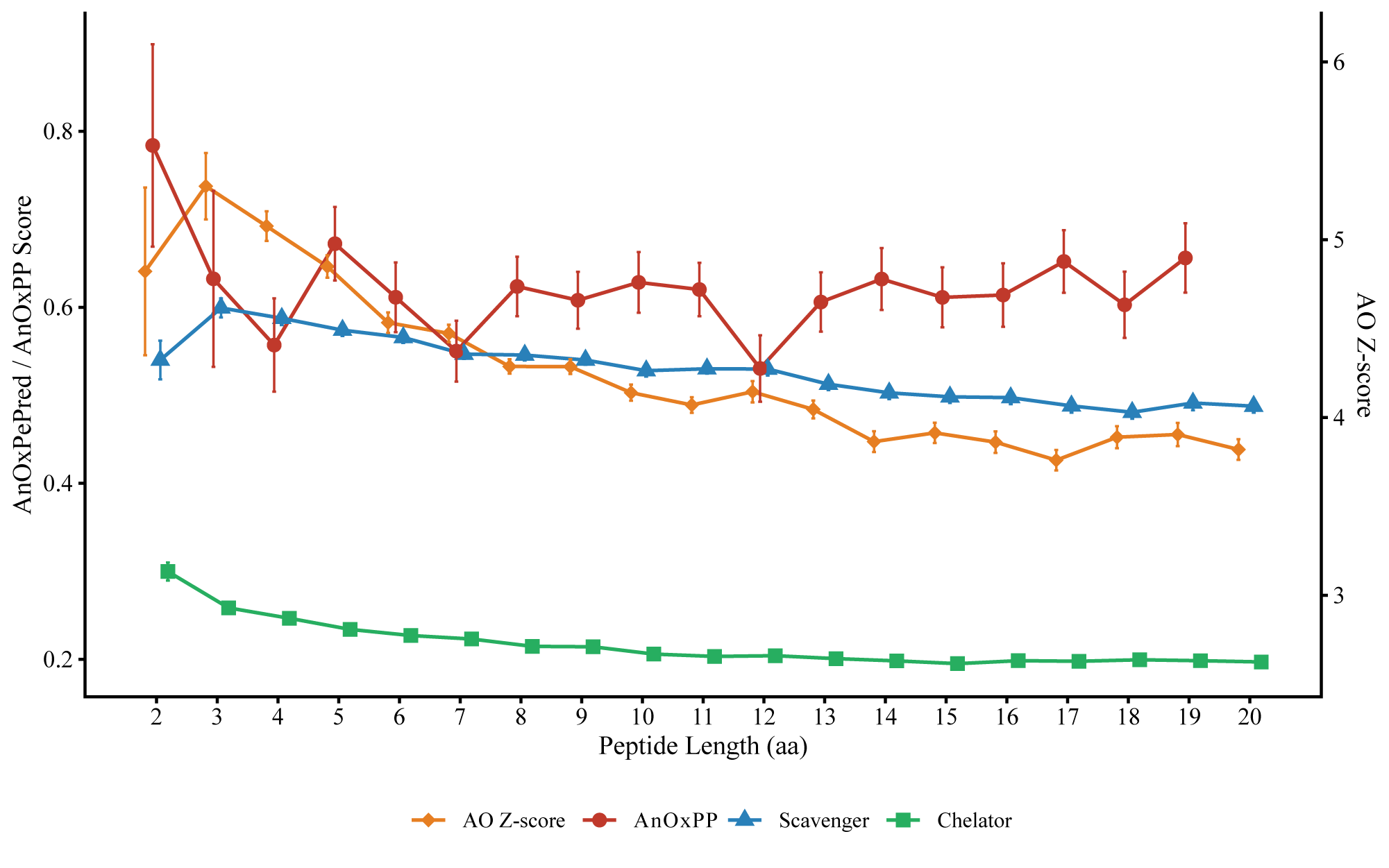

Supplement: SUPPLEMENTARY FIGURE S4 — Length-dependent antioxidant prediction scores. Mean scores (±SE) for peptides of 2–20 amino acids. Short peptides (2–5 aa) showed higher AnOxPP and AO Z-scores, while Scavenger scores remained stable and Chelator scores were uniformly low across all lengths. [file Image_4.TIF]
